# Supplementary material for: Assessment of Antibody-Titer Changes after Second and Third Severe Acute Respiratory Syndrome Coronavirus 2 mRNA Vaccination in Japanese Post-Kidney-Transplant Patients
Source: Vaccines (Basel). 2023 Jan 6;11(1):134. doi: 10.3390/vaccines11010134 (PMC9866315; doi:10.3390/vaccines11010134)
Supplement: Supplementary file 1 [file vaccines-11-00134-s001.zip › vaccines-2022976-supplementary.pdf]

Supplemental table S1. N-IgG titers in patients with elevated S-IgG titers at 6 months after the second vaccination

	S-IgG (AU/mL)			N-IgG (AU/mL)
	**Second vaccination**	**6 months after second vaccination**	**Third vaccination**	
**Patient 1**	37.4	51.5	3050	0.02
**Patient 2**	16.5	350.9	6790	0.12
**Patient 3**	32.2	92.7	21900	0.01
